# PHYSICAL ATTRIBUTES LINKED TO FRAILTY IN NURSING HOME RESIDENTS: INSIGHTS FROM A CROSS-SECTIONAL STUDY

**DOI:** 10.1093/geroni/igae098.4044

**Published:** 2024-12-31

**Authors:** Francis Kin Tung Chan, Chi Tat Fung, Ka Po Chan, Yijian Yang

**Affiliations:** Hong Kong Sheng Kung Hui Welfare Council Limited, Hong Kong, Hong Kong, China (People’s Republic); Hong Kong Sheng Kung Hui Welfare Council Limited, Hong Kong, Hong Kong, China (People’s Republic); The Chinese University of Hong Kong, Hong Kong, China (People’s Republic); The Chinese University of Hong Kong, Hong Kong, China (People’s Republic)

## Abstract

Frailty is a complex geriatric condition characterized by reduced physiological reserve and increased risk of adverse outcomes. Older adults in nursing homes are generally frail and have poor mobility. However, research on frailty in nursing homes is scarce compared to community settings, particularly on its association with mobility and postural stability. A cross-sectional analysis of baseline data from a two-arm cluster randomized controlled trial was conducted, involving 133 participants aged 60 and above from 20 Hong Kong residential care homes. The FRAIL-NH scale was used to assess frailty through factors such as fatigue, mobility, and nutrition for nursing home residents. Walking speed was assessed by the GAITRite computerized walkway system, postural stability with the Swaymeter. Multiple regression statistical analysis was conducted to identify associations between FRAIL-NH and physical attributes. Walking speed (Standardized Coefficients = -0.38, p< 0.001), anterior-posterior body sway (Standardized Coefficients = -0.288, p=0.02), and BMI (Standardized Coefficients = -1.98, p=0.018) were significantly associated with FRAIL-NH, with walking speed as the strongest predictor account for 19.6% of total variance. However, medial-lateral body sway did not show significant association with FRAIL-NH. Additionally, no significant correlation was observed between specific muscle strength measures and FRAIL-NH. Our study highlights walking speed and anterior-posterior sway as key indicators of frailty in nursing home residents. These results identify the need for focused interventions that improve balance and mobility, which may potentially decrease frailty and risk of falling for this vulnerable population. Further research is crucial for developing effective frailty interventions in nursing homes.

